# Clinical Characteristics and Risk Factors for Cryptococcal Meningitis in Diverse Patient Populations in New York City

**DOI:** 10.1093/ofid/ofae576

**Published:** 2024-10-09

**Authors:** Zomer Sardar, Carla Y Kim, Kiran T Thakur

**Affiliations:** Department of Neurology, Columbia University Irving Medical Center, New York, New York, USA; Department of Neurology, Mayo Hospital, Lahore, Punjab, Pakistan; Department of Neurology, Columbia University Irving Medical Center, New York, New York, USA; Department of Neurology, Columbia University Irving Medical Center, New York, New York, USA

**Keywords:** ART adherence, cryptococcal meningitis, HIV, immunocompromised, transplant

## Abstract

**Background:**

Cryptococcal meningitis (CM) is responsible for 15%–20% of human immunodeficiency virus (HIV)–associated mortalities. CM prevalence has also increased in other immunocompromised populations of transplant recipients, patients with cancer, and individuals on immunomodulatory medication.

**Methods:**

This retrospective review included 51 definitive patients with CM hospitalized at a tertiary academic medical center in New York City between 2010 and 2023. We assessed clinical features and outcomes of CM, with additional analysis of factors related to antiretroviral therapy (ART) adherence in HIV-infected cases and immunomodulatory medication history of HIV-negative cases.

**Results:**

The cohort had a mean (standard deviation) age of 47.1 ± 15.1 years, and was predominantly male (37, 72.5%). Of 32 patients with HIV, 3 (9.4%) were newly diagnosed with HIV at the time of CM hospitalization, 5 (15.6%) had recurrent CM, and 2 (6.3%) had a CM relapse. The majority (30, 93.8%) of patients with HIV were ART nonadherent. Of 19 HIV-negative patients, 8 (42.1%) were solid-organ transplant recipients, 5 (26.3%) had autoimmune conditions of sarcoidosis or systemic lupus erythematosus, and 3 (15.8%) had chronic lymphocytic leukemia. Six (11.8%) patients died during hospitalization, 4 of whom had HIV.

**Conclusions:**

The burden of CM in people with HIV and immunocompromised patients continues even in settings with accessible standard antifungal treatment though interventions of increased ART adherence for those with HIV and antifungal prophylaxis may improve morbidity and mortality.

## INTRODUCTION

Despite advances in prevention, diagnosis and treatment for cryptococcal meningitis (CM), the condition remains the most common human immunodeficiency virus (HIV)-associated central nervous system infection, implicated in 15%–20% of all HIV-related deaths [1, 2]. In the United States, there are an estimated 3400 new cases of CM annually with up to 700 deaths per year [3]. In addition to the ongoing burden of disease in people with HIV (PWH), CM is increasingly prevalent in patients immunocompromised after solid organ transplantation, individuals with malignancy, and the expanding population on chronic immunomodulatory medicines [4–6]. In resource-rich settings, HIV-negative CM accounts for at least 25% of all CM-related hospitalizations and deaths and as many as 30% of HIV-negative CM cases had no prior underlying conditions [7, 8]. In this analysis, we aim to characterize the risk factors, clinical features, and outcomes of CM in populations with and without HIV at a tertiary medical academic center in New York City. This study also evaluates factors influencing nonadherence to antiretroviral therapy (ART) in the cohort of those with HIV and CM.

## MATERIALS AND METHODS

**Figure 1.**
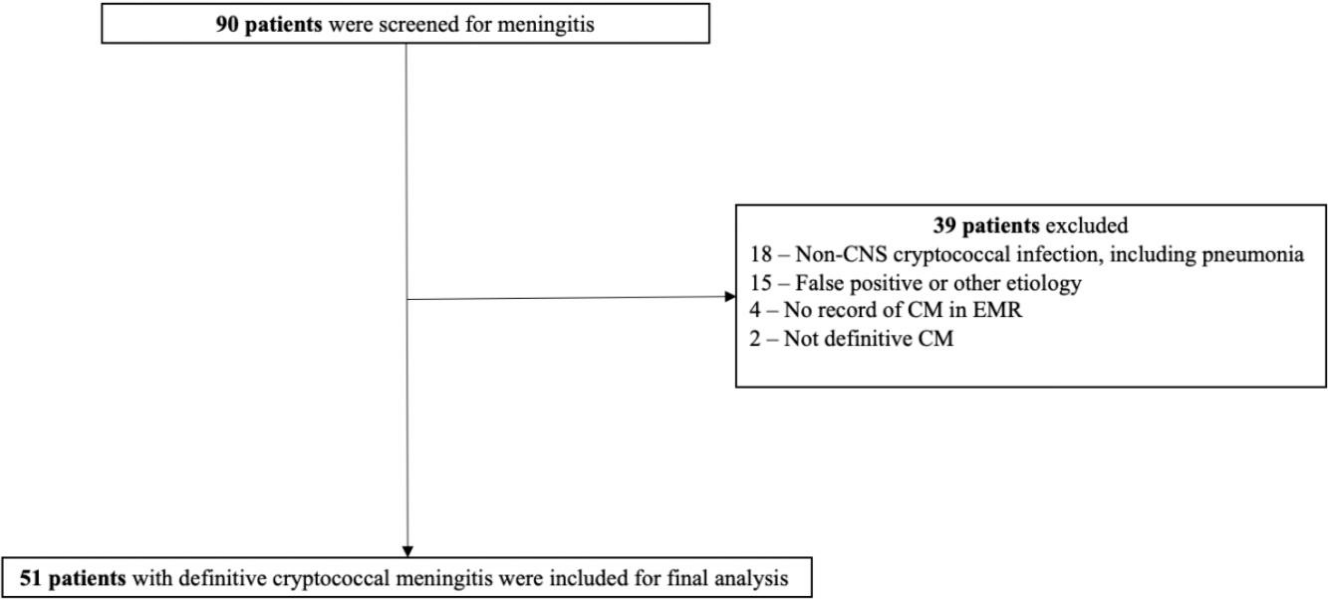
Flowchart of cryptococcal meningitis cases.

A retrospective review of electronic medical records, obtained from the microbiology laboratory and bioinformatics department of a large tertiary academic medical center, was conducted of hospitalized patients with CM between 2010 and 2023. Patients met criteria for definitive CM by 1 of the following diagnostic methods: (1) growth of *Cryptococcus neoformans* in cerebrospinal fluid (CSF) culture, (2) positive polysaccharide cryptococcal antigen in CSF [9]. Relapse of CM was defined as the recovery of *Cryptococcus neoformans* or *Cryptococcal gattii* in CSF culture after a negative CSF culture subsequent to antifungal therapy from previous infection [10]. Recurrence of CM was defined as recurrence of signs and symptoms in the setting of persistent infection [11]. Immunocompromised status was defined as immunocompromised because of HIV (group with HIV) and immunocompromised because of organ transplantation, hematological malignancy, immunomodulatory or immunosuppressive therapy, or reason unknown (HIV-negative group) [12]. In the group with HIV, patients were classified as newly diagnosed if they did not have a record of positive HIV status or not initiated on highly active ART. The screening, eligibility, and enrollment of patients with CM is outlined in Figure 1.

Sociodemographic features, clinical data including neuroimaging and CSF findings, history of immunomodulatory treatment, immunosuppressed status including factors associated with antiretroviral use in PWH (adherence to ART, reasons of nonadherence to ART, barriers to HIV care), length of hospital stay, time to initiation of treatment after admission, time to occurrence of CM after organ transplant, and outcomes defined by death and discharge from hospital were obtained.

### Statistical Analysis

Descriptive analysis of demographic and temporal clinical variables of age, CD4 count, CSF parameters, and length of hospital stay, time from clinical presentation to diagnosis and initiation of antimicrobial treatment are presented as mean, median, and standard deviations. Social determinants of health and clinical factors of symptom presentation, imaging abnormalities, drug use, immune status, treatment, treatment-related complications, and outcomes are presented as frequency and percentages. All data were entered and analyzed in SPSS 24.0, IBM (Chicago, IL, USA).

### Patient Consent Statement and Ethical Clearance

This retrospective descriptive study received a waiver of consent and was approved by the institution review board and ethical committee of Columbia University Irving Medical Center-New York Presbyterian Hospital, New York, USA (IRB# AAAR3440).

## RESULTS

### Demographics

Fifty-one patients with definitive CM were included in the study. The mean age of the patients was 47.1 ± 15.1 years and they were predominantly male (37, 72.5%) and non-Hispanic (24, 47.1%). There were 3 (5.9%) immunocompetent patients and 48 (94.1%) immunocompromised patients. Of the 48 immunocompromised CM patients, 32 (66.7%) were HIV-positive.

The mean age in the group with HIV group was 41.8 ± 11.5 years, 23 (71.9%) identified as male, 3 (9.4%) identified as transgender female, and 13 (40.6%) identified as Black/African-American. In the HIV-negative group, the mean age was 52.3 ± 18.3 years, with 14 (73.7%) males and 5 (26.3%) females. Five (26.3%) identified as White, 4 (21.1%) identified as Black/African American, and 3 (15.8%) identified as Asian. In the group with HIV at the time of presentation, 9 (28.1%) patients were unemployed and 7 (21.9%) were homeless with no access to stable housing. There were no homeless patients in the HIV-negative group, 12 (63.2%) of whom were living with a spouse or partner and 16 (84.2%) living independently in a single or multiple private residence (Table 1).

**Table 1.** Demographics and Social Determinants of Health of Cohort

	Total Cohort	HIV-positive	HIV-negative
	N = 51	N = 32	N = 19
Demographics			
Mean age ± SD (range)	45.7 ± 15.1 (20–88)	41.8 ± 11.5 (24–73)	52.3 ± 18.3 (20–88)
Self-identified gender			
Male	37 (72.5%)	23 (71.9%)	14 (73.7%)
Female	11 (21.6%)	6 (18.8%)	5 (26.3%)
Female transgender	3 (5.9%)	3 (9.4%)	0
Self-identified race			
Black/African/American	17 (33.3%)	13 (40.6%)	4 (21.1%)
White	8 (15.7%)	3 (9.4%)	5 (26.3%)
Asian	3 (5.9%)	0	3 (15.8%)
Other Pacific Islander	6 (11.85)	6 (18.8%)	0
Unknown/declined	17 (33.3%)	10 (31.3%)	7 (36.8%)
Self-identified ethnicity			
Hispanic/Latinx	12 (23.5%)	10 (31.3%)	2 (10.5%)
Non-Hispanic/Latinx	24 (47.1%)	13 (40.6%)	11 (57.9%)
Unknown/declined	15 (29.4%)	9 (28.1%)	6 (31.6%)
Marital status			
Single	29 (56.9%)	25 (78.1%)	4 (21.1%)
Married	17 (33.3%)	4 (12.5%)	13 (68.4%)
Divorced	3 (5.9%)	2 (6.3%)	1 (5.3%)
Unknown	2 (3.9%)	1 (3.1%)	1 (5.3%)
Highest education level completed			
High school/GED	6 (11.8%)	4 (12.5%)	2 (10.5%)
Some college/AA/tech school	4 (7.8%)	16 (50.0%)	1 (5.3%)
Master's degree	1 (2.0%)	0	1 (5.3%)
Unknown/declined	40 (78.4%)	25 (78.1%)	15 (78.9%)
Employment			
Yes	17 (33.3%)	9 (28.1%)	8 (42.1%)
No	23 (45.1%)	16 (50.0%)	7 (36.8%)
Unknown	11 (21.6%)	7 (21.9%)	4 (21.1%)
Preferred language			
English	39 (76.5%)	27 (84.4%)	12 (63.2%)
Spanish	5 (9.8%)	4 (12.5%)	1 (5.3%)
Other	6 (11.8%)	0	6 (31.6%)
Unknown	1 (2.0%)	1 (3.1%)	0
Social determinants of health			
Homelessness			
Yes	7 (13.7%)	7 (21.9%)	0
No	39 (76.5%)	20 (62.5%)	19 (100%)
Unknown	5 (9.8%)	5 (15.6%)	0
Living situation			
Lives alone	10 (19.6%)	8 (25.0%)	2 (10.5%)
Lives with 1 person: spouse or partner	17 (33.3%)	5 (15.6%)	12 (63.2%)
Lives with 1 person: relative, friend, roommate	8 (15.7%)	7 (21.9%)	1 (5.3%)
Lives with caregiver: not spouse, partner, relative, friend, roommate	1 (2.0%)	0	1 (5.3%)
Lives with group in a private residence	3 (5.9%)	2 (6.3%)	1 (5.3%)
Lives in group home: assisted living, nursing home	4 (7.8%)	3 (9.4%)	1 (5.3%)
Unknown	8 (15.7%)	7 (21.95)	1 (5.3%)
Level of independence			
Able to live independently	39 (76.5%)	25 (78.1%)	16 (84.2%)
Requires some assistance with basic activities	2 (3.9%)	0	0
Requires assistance with complex activities	2 (3.95)	2 (6.3%)	1 (5.3%)
Completely dependent	0	0	0
Unknown	8 (15.7%)	5 (19.2%)	2 (10.5%)
Housing type			
Single or multiple-family private residence	26 (51.0%)	12 (43.8%)	12 (63.2%)
Independent group living	1 (2.0%)	1 (3.1%)	0
Assisted living, adult family home, boarding home, SRO	3 (5.9%)	4 (3.8%)	0
Skilled nursing facility, nursing home, hospice	2 (3.9%)	1 (3.1%)	1 (5.3%)
Unknown	19 (37.3%)	…	6 (31.6%)
Has primary care provider			
Yes	37 (72.5%)	23 (71.9%)	13 (68.4%)
No	13 (25.5%)	9 (28.1%)	6 (31.6%)

Abbreviations: AA, associate of arts; SD, standard deviation; SRO, single room occupancy.

### Clinical Course

Headache was the most frequent clinical symptom in both HIV-positive (15/32; 46.9%) and HIV-negative (10/19; 52.6%) cohorts, followed by altered mental status (5/32 in HIV-positive; 5/19 in HIV-negative) and seizures (3/32 in HIV-positive and 3/19 in HIV-negative). Neuroimaging findings such as hydrocephalus (5, 26.3%), infarcts (5, 26.3%), and meningeal enhancement (4, 21.1%) were more frequent in the HIV-negative group. A ventriculoperitoneal shunt was placed in 3/19 (15.8%) patients in the HIV-negative group, and lumbar drain was placed in 1/32 (3.1%) patients in the HIV group (Table 2).

**Table 2.** Clinical Presentation, Cerebrospinal Fluid Studies, Radiographic Data, and Treatment of Cohort

	Total Cohort	HIV-positive	HIV-negative
	N = 51	N = 32	N = 19
Clinical Presentation			
Headache			
Yes	25 (49.0%)	15 (46.9%)	10 (52.6%)
No	15 (29.4%)	9 (28.1%)	6 (31.6%)
Unknown	11 (21.6%)	8 (25.0%)	3 (15.8%)
Seizures			
Yes	6 (11.8%)	3 (9.4%)	3 (15.8%)
No	34 (66.7%)	22 (68.8%)	12 (63.2%)
Unknown	11 (21.6%)	6 (18.8%)	4 (21.1%)
Limb weakness			
Yes	5 (9.8%)	0	3 (15.8%)
No	35 (68.6%)	25 (78.1%)	10 (63.2%)
Unknown	7 (13.7%)	7 (21.9%)	4 (21.1%)
CSF studies			
Mean CSF WBC Count ± SD (Range)	126.7 ± 202.17 (0–791)	70.8 ± 162.0 (0–734)	215.94 ± 230.6 (2–791)
Mean CSF Glucose ± SD (range), mg/dL	48.2 ± 25.36 (5–128)	48.50 ± 20.94 (17–128)	47.73 ± 31.76 (5–125)
Mean CSF protein ± SD (range), mg/dL	108.93 ± 118.0 (17–682)	87.17 ± 85.17 (17–388)	143.31 ± 153.1 (23–682)
Mean LP opening pressure ± SD (range), mm Hg	26.2 ± 11.9 (8–55)	28.6 ± 10.4 (9–50)	23.6 ± 13.6 (8–55)
Radiographic findings			
Findings			
Hydrocephalus	7 (13.7%)	2 (6.3%)	5 (26.3%)
Infarct	7(13.7.%)	2 (6.3%)	5 (26.3%)
Enhancement	6 (11.8%)	2 (6.3%)	4 (21.1%)
Shunt/lumbar drain	5 (9.8%)	2 (6.3%)	3 (15.8%)
Treatment			
Amphotericin	4 (7.8%)	3 (9.4%)	1 (5.3%)
Amphotericin + flucytosine	38 (74.5%)	21 (65.6%)	16 (84.2%)
Amphotericin + fluconazole	2 (3.95)	2 (6.3%)	1 (5.3%)
High-dose fluconazole	1 (2.0%)	1 (3.1%)	0
Fluconazole + flucytosine	1 (2.0%)	1 (3.1%)	0
Amphotericin + flucytosine + voriconazole	1(2.0%)	1(3.1%)	0
Unknown	5 (9.8%)	3 (9.4%)	1 (5.3%)

Abbreviations: CSF, cerebrospinal fluid; LP, lumbar puncture; SD, standard deviation; WBC, white blood cell.

Data on treatment were available for 29 individuals in HIV-positive patients. Amphotericin and flucytosine were provided to 21 (72.4%) patients in the HIV-positive cohort. One (3.4%) received high-dose fluconazole (800 mg/day), 1 (3.4%) received amphotericin in combination with flucytosine and voriconazole, and 1 (3.4%) received amphotericin with fluconazole. Three (10.3%) patients were treated with amphotericin only, with no clarifying explanation in the case notes. In the HIV-negative group, treatment records were available in 17 (89.5%) patients, of which 16 (84.2%) received a combination of amphotericin and flucytosine. Cryptococcal meningitis prophylaxis data were only available for 5 patients in the HIV-positive group, of which only 1 (3.4%) patient was adherent to prophylactic therapy (Table 2).

Five (15.6%) patients in the HIV-positive group had a recurrence of CM and 2 (6.3%) patients had a relapse of CM. Amphotericin-related adverse effects (9/32) were reported in the HIV-positive group, 3 (9.4%) patients had acute kidney injury, 1 (3.1%) patient had hypokalemia, 1 (3.1%) patient had fever after administration of amphotericin, and 1 (3.1%) had a rash (red man syndrome). In the HIV-negative group, 3 (15.8%) patients had acute kidney injury secondary to amphotericin, and bone marrow suppression (pancytopenia) was observed in 2 (10.5%) secondary to flucytosine. One (5.3%) patient had experienced adverse effects to both drugs (acute kidney injury from amphotericin; pancytopenia from flucytosine). *Pneumocystis jirovecii* pneumonia 10 (31.3%) and oral candidiasis 9 (28.1%) were found to be the most common concomitant opportunistic infections in CM patients in the HIV group, and in HIV-negative group 1 (5.3%) had *P jirovecii*.

### ART Adherence and CD4 Count in HIV-positive Cohort

Of 32 HIV-positive patients, 3 (9.4%) were newly diagnosed with HIV and 30 (93.8%) subjects were not adherent to their ART regimen. Reasons for nonadherence were determined by chart review in 20 (62.5%) patients. Insurance issues with an inability to pay for medications were reported by 4 (12.5%), 1 (3.1%) patient reported that their medications were stolen, 1 (3.1%) patient discontinued ART because they felt their medication burden was too high, 1 (3.1%) patient stopped taking ART because of concerns around other comorbid conditions, 1 (3.1%) patient reported transportation problems to obtain medications, 1 (3.1%) patient had a fear of swallowing tablets, 1 (3.1%) patient felt shame about her diagnosis of HIV and therefore was not taking ART regularly, and 1 (3.1%) patient reported “family issues” as a cause of nonadherence to ART (Table 3).

**Table 3.** ART Adherence and CD4 Count in HIV-positive Cohort

HIV-positive Cohort	
N = 32	
CD4 count	
<100	29 (90.6%)
100–500	2 (6.3%)
>500	1 (3.1%)
Antiretroviral therapy adherence	
Adherence	2 (6.3%)
Nonadherence	30 (93.8%)
Unknown	0
Reasons for nonadherence	
Insurance issues	4 (12.4%)
Newly diagnosed	3 (9.4%)
Liver issues	1 (3.1%)
Pill burden	1 (3.1%)
Transportation issues	1 (3.1%)
Shame	1 (3.1%)
Stolen/sold medication	1 (3.1%)
Family issues	1 (3.1%)
Fear of swallowing pills	1 (3.1%)
Unknown	12 (37.5%)
Missed appointments in last 12 mo	
Yes	11 (34.3%)
No	2 (6.3%)
Unknown	15 (57.7%)
HIV drug resistance (nonnucleoside reverse transcriptase inhibitor)	2 (6.3%)

Of 32 HIV-positive patients, 1 (3.1%) had a CD4 count > 500 per mm^3^, undetectable viral load, and was taking ART intermittently because of hepatitis-induced liver cirrhosis with a history of transhepatic intrajugular portosystemic shunting. One (3.1%) patient had a relapse of CM, and 4 (12.5%) patients had a recurrence of CM, all of whom ART nonadherent and additionally, noncompliant with fluconazole if prescribed previously as consolidation or maintenance therapy (Table 3).

### Immunocompromised Conditions in the HIV-negative Group

Eight (42%) of the patients in the HIV-negative group with CM were on immunosuppression after organ transplantation, most commonly kidney transplant (5, 26.3%). One patient with a liver transplant received tacrolimus and oral prednisone. The median duration of onset of CM after transplant was 2 (interquartile range, 1–4.25) years. Immunosuppressive drugs used for recipients of solid organ transplants can be seen in Table 4.

**Table 4.** Immunocompromised Status and Medication for HIV-negative Cohort

HIV-negative Cohort N = 19	
CLL	3 (15.8%)
Organ transplantation	8 (42.1%)
Kidney	5/8
Lung	2/8
Liver	1/8
Autoimmune disease	5 (26.3%)
SLE	2/5
Sarcoidosis	2/5
Neurosarcoidosis	1/5
Immunosuppressants	1 (5.3%)
Immunocompetent	3 (15.8%)

Sex/Age	Condition	Immunosuppressant Medication	Intervention	Time to Development of Cryptococcal Infection After Immunocompromised Status
M63	CLL, small cell lymphoma	Lenalidomide, rituximab	NA	1 y after transplant
M75	CLL	Ibrutinib	NA	7 y after diagnosis of CLL
M57	CLL	Ibrutinib, Obinutuzumab	NA	1 y after diagnosis of CLL
M44	Lung transplant	Tacrolimus, oral prednisone	NA	3 y after transplant
M64	Lung transplant	Mycophenolate mofetil, oral prednisone	NA	1 y after transplant
M64	Liver transplant	Tacrolimus, oral prednisone	NA	5 y after transplant
M63	Kidney transplant	Cyclosporin, oral prednisone	NA	1 y after transplant
F45	Kidney transplant	Oral prednisone, eculizumab trial	NA	2 y after transplant
F47	Kidney transplant	Graft failure, oral prednisone, tacrolimus	VPS	1 y after transplant
M60	Kidney transplant with ESRD	Tacrolimus	VPS Deceased	4 y after transplant
M74	Kidney transplant	Tacrolimus, oral prednisone, Leflunomide	NA	2 y after transplant
M44	Sarcoidosis in 2003	Oral prednisone	NA	10 y after diagnosis of sarcoidosis
M41	Sarcoidosis	Oral prednisone	NA	6 y after diagnosis of sarcoidosis
M35	Sarcoidosis with CNS involvement	Oral prednisone	Extraventricular drain followed by VPS	4 y after diagnosis of sarcoidosis
F25	SLE, APLS, ARF	Oral prednisone, mycophenolate mofetil, cyclophosphamide, rituximab	NA	5 y after diagnosis of SLE

Abbreviations: APLS, antiphospholipid syndrome; ARF, acute renal failure; CLL, chronic lymphocytic leukemia; CNS, central nervous system; ESRD, end-stage renal disease; NA, not available; SLE, systemic lupus erythematosus; VPS, ventriculoperitoneal shunting.

Of the 5 (26.3%) patients with autoimmune conditions, 2 (5.3%) had systemic lupus erythematosus, 3 (15.8%) had sarcoidosis, 1 of whom had sarcoidosis with central nervous system involvement, and 1 (5.3%) patient was on mycophenolate mofetil (MMF) but the diagnosis was not reported. One patient with systemic lupus erythematosus and antiphospholipid syndrome (APLA) was prescribed oral prednisone, MMF, cyclophosphamide, and rituximab. The 1 neurosarcoidosis and 2 sarcoidosis patients were on oral prednisone. The patient with neurosarcoidosis was also on oral prednisone. The diagnosis was not mentioned for the last patient but it was noted that they were taking MMF. Of the 3 (15.8%) patients with chronic lymphocytic leukemia, 2 were taking ibrutinib and 1 was also prescribed obinutuzumab and ibrutinib. The third patient with chronic lymphocytic leukemia and small cell lymphoma was taking rituximab and lenalidomide (Table 4).

There were 3 (15.8%) patients with negative workup for immunocompromised state. The cause of CM in 1 patient, a 20-year-old male, was attributed to parakeet exposure (Table 4).

Four (12.5%) patients with HIV and 1 (5.3%) HIV-negative patient died during hospitalization. The cause of death in all patients was multiorgan failure and secondary septic shock related to multiple opportunistic infections including CM in HIV-positive patients and disseminated cryptococcus in the HIV-negative patient. Detailed outcomes of patients with HIV-positive and HIV-negative related CM are outlined in Table 5.

**Table 5.** Discharge Disposition and Outcomes of Cohort

Discharge Disposition			
	Total Cohort N = 51	HIV N = 32	HIV-negative N = 19
Home	22 (43.1%)	15 (46.9%)	7 (36.8%)
Home with health service	3 (5.9%)	2 (6.3%)	1 (5.3%)
Skilled nursing facility	3 (5.9%)	1 (3.1%)	2 (10.5%)
Rehabilitation facility	7 (13.7%)	3 (9.4%)	4 (21.1%)
Left against medical advice	2 (3.9%)	2 (6.3%)	0
Unknown	5 (9.8%)	4 (12.5%)	1 (5.3%)

Characteristics of Deceased Patients					
HIV	Patients	CD4 count per mm^3^	ART Adherence	Concomitant Opportunistic Infections	Cause of Death
	39 y/M	<100/mm^3^	No	PCP, Cryptosporidium, oral *Candida*	MOF
	29 y/M	<100/mm^3^	No	*Mycobacterium avium* complex, Kaposi sarcoma	MOF, septic shock
	35 y/M	<100/mm^3^	No	Oral candidiasis, PCP, disseminated cryptococcus with meningitis, CMV, TB	MOF, Septic shock
	32 y/F Transgender	<100/mm^3^	No	Multiple infections but records unclear	Septic shock

HIV-negative	Patients	PMH	Clinical Factors	Cause of Death
	75 y/M	CLL	Disseminated Cryptococcus ibrutinib	Septic shock

Abbreviations: CLL, chronic lymphocytic leukemia; CMV, cytomegalovirus; MOF, multiorgan failure; PCP, pneumocystis pneumonia; PMH, past medical history; TB, tuberculosis.

## DISCUSSION

Cryptococcal meningitis remains the leading cause of morbidity and mortality in advanced cases of HIV despite existing therapeutics for prevention and treatment, accounting for approximately 20% of all HIV-related deaths [13]. Inadequate access to preventive screening and first-line therapeutics in resource-limited settings correspond to 20% survival rates, according to the global initiative from South Africa and the Global Action for Fungal Infections, “Ending Cryptococcal Meningitis Deaths by 2030 Strategic Framework” [14]. Even in developed countries where cryptococcal antigen and CD4+ screening are widely available, mortality in cryptococcal meningoencephalitis ranges between 6% and 16% [15]. Although ART has decreased the global incidence of HIV, ART adherence is the mainstay of prevention of opportunistic infections because it not only decreases morbidity and mortality, but also decreases the emergence of resistant strains of HIV [16]. Compliance with ART is also a strong predictor of CM treatment success in PWH [10].

The primary risk factor for CM is acquired immunodeficiency syndrome, particularly in individuals with a CD4 count below 100 mm^3^. In HIV-negative individuals, other notable risk factors include glucocorticoid therapy, sarcoidosis, and idiopathic CD4 lymphopenia [6]. Additionally, case reports and case series have identified several other risk factors, including the use of anti–tumor necrosis factor-α agents such as infliximab, anti-CD52 agents like alemtuzumab, Bruton tyrosine kinase inhibitors such as ibrutinib, and sphingosine-1-phosphate receptor modulators including fingolimod, tacrolimus, and sirolimus [6]. The majority of our patients in the HIV-negative group were solid organ transplant recipients and 5 patients with autoimmune conditions who were on immunosuppressant and immunomodulatory therapies.

The reasons for ART non-adherence vary among patients with HIV, some of which relate to food security, lack of employment, lack of insurance, high volume of medication, stigma related to HIV diagnosis, malnutrition, education status, forgetfulness, and fear of being judged in public or at the workplace [17–19]. In our study, a significant number (92.3%) of patients were noncompliant with ART with reasons similar to those identified in prior studies. Barriers to ART and patients’ concerns related to medication, adverse outcomes, pill burden, and complicated regimens should be addressed with education and counseling about the risks of nonadherence on initiation of treatment. In those tested, we observed nonnucleoside reverse transcriptase inhibitors resistance in 2 (6.3%) of our patients and both were nonadherent to the ART medication.

In addition to PWH, the epidemiology of CM has been altered by immunomodulatory therapies rendering solid-organ transplant, cancer chemotherapy recipients, and patients with autoimmune conditions vulnerable to cryptococcal infection, resulting in approximately one-third of CM-related deaths attributable to HIV-negative populations, particularly in high-resource settings [6]. The mortality rate of CM was found to be comparable between HIV-negative and HIV-positive patients, with long-term cognitive, motor, visual, and hearing impairment in 20%–70% of survivors at 1 year after infection [20]. *Cryptococcal neoformans* infection is ranked third among recipients of solid organ transplants, with a prevalence of 25% to 72% [21, 22]. CM usually manifests as a late-occurring infection with a median range of CM diagnosis of 16–24 months after solid organ transplantation, across 4 studies [23–27]. Among our cases with solid organ transplants, the median time to onset of CM after solid transplantation was approximately 2 (interquartile range, 1–4.25) years.

Prior studies have reported headache and altered mental status as the most common presenting symptoms among patients with HIV-negative cryptococcal meningitis [28]. Although headache was frequently reported in both our groups, altered mental status was less prominent in our study, which could be an effect of the small sample size. Imaging abnormalities in CM typically includes infarction, leptomeningeal enhancement, and hydrocephalus, with cerebral ischemia observed in 13%–21.5% of patients with CM [29]. Most of the infarctions were reported to be lacunar in our cohort and we observed cerebral ischemia in the basal ganglia in 7 (13.7%) of our patients. Other studies have reported lacunar type infarction as the most common infarction type, with the basal ganglia as the most common site of infarction in 57.2% of the patients with CM [30]. The difference in CSF results was also observed in HIV-positive and HIV-negative subgroups. CSF cell count in patients with HIV was lower, ranging from 0 to 5 cells/μL in 37% (12/32) individuals, whereas CSF cell count was higher in HIV-negative patients (20–200 cells/μL) [31].

The gold standard treatment for induction therapy in patients with cryptococcal meningoencephalitis is amphotericin B with flucytosine for 2 weeks followed by 8 weeks of fluconazole [14, 32, 33]. In our study, 5/32 HIV-positive patients did not complete induction therapy for unclear reasons. The relapse of CM is associated with primary inadequate therapy or nonadherence to fluconazole consolidation or maintenance therapy [11]. In our study, 2 patients in the HIV-positive group had a relapse of CM.

This study has several limitations including its retrospective design and reliance on electronic medical records with missing data. All cases were identified from a single academic tertiary center, limiting generalizability of findings. Because of the low statistical power of the small cohort size, only descriptive analysis was performed. However, we characterized all definitive CM cases with granularity over a 13-year span in a diverse and densely populated region of the United States, highlighting the evolving epidemiology of CM cases across different immunocompromised populations at a large medical center. Our study findings echo those of similar cohort studies from other US regions in the highly active ART era [34, 35].

Cryptococcal meningoencephalitis remains a significant issue in PWH and immunocompromised patients, even in resource-rich regions. Large-scale, multicenter, longitudinal studies should be conducted to track epidemiological shifts in CM-vulnerable populations, and to prospectively address socioeconomic barriers that obstruct the continuum of care, ART adherence, early diagnosis, and prompt initiation of antifungal treatment.
